# Author Correction: CD201^+^ fascia progenitors choreograph injury repair

**DOI:** 10.1038/s41586-023-06928-2

**Published:** 2023-12-06

**Authors:** Donovan Correa-Gallegos, Haifeng Ye, Bikram Dasgupta, Aydan Sardogan, Safwen Kadri, Ravinder Kandi, Ruoxuan Dai, Yue Lin, Robert Kopplin, Disha Shantaram Shenai, Juliane Wannemacher, Ryo Ichijo, Dongsheng Jiang, Maximilian Strunz, Meshal Ansari, Illias Angelidis, Herbert B. Schiller, Thomas Volz, Hans-Günther Machens, Yuval Rinkevich

**Affiliations:** 1 Institute of Regenerative Biology and Medicine (IRBM), Helmholtz Munich, Munich, Germany; 2 Member of the German Centre for Lung Research (DZL), Comprehensive Pneumology Center (CPC) and Institute of Lung Health and Immunity (LHI), Helmholtz Munich, Munich, Germany; 3 Institute of Experimental Pneumology, Ludwig-Maximilians University Hospital, Munich, Germany; 4 Klinikum rechts der Isar, Department of Dermatology, School of Medicine, Technical University of Munich, Munich, Germany; 5 Klinikum rechts der Isar, Department of Plastic and Hand Surgery, School of Medicine, Technical University of Munich, Munich, Germany

**Keywords:** Differentiation, Stem-cell research

Correction to: *Nature* 10.1038/s41586-023-06725-x Published online 15 November 2023

In the version of the article initially published, the surname of Thomas Volz appeared incorrectly as Voltz. This has now been corrected in the HTML and PDF versions of the article.

