# Supplementary material for: CD201+ fascia progenitors choreograph injury repair
Source: Nature. 2023 Nov 15;623(7988):792–802. doi: 10.1038/s41586-023-06725-x (PMC10665192; doi:10.1038/s41586-023-06725-x)
Supplement: Supplementary file 1 — Reporting Summary [file 41586_2023_6725_MOESM1_ESM.pdf]

nature portfolio

Corresponding author(s): Yuval Rinkevich

Last updated by author(s): Sep 27, 2023

Reporting Summary

Nature Portfolio wishes to improve the reproducibility of the work that we publish. This form provides structure for consistency and transparency in reporting. For further information on Nature Portfolio policies, see our Editorial Policies and the Editorial Policy Checklist.

Statistics

For all statistical analyses, confirm that the following items are present in the figure legend, table legend, main text, or Methods section.

n/a	Confirmed	
☐	☒	The exact sample size (n) for each experimental group/condition, given as a discrete number and unit of measurement
☐	☒	A statement on whether measurements were taken from distinct samples or whether the same sample was measured repeatedly
☐	☒	The statistical test(s) used AND whether they are one- or two-sided<br>*Only common tests should be described solely by name; describe more complex techniques in the Methods section.*
☒	☐	A description of all covariates tested
☐	☒	A description of any assumptions or corrections, such as tests of normality and adjustment for multiple comparisons
☐	☒	A full description of the statistical parameters including central tendency (e.g. means) or other basic estimates (e.g. regression coefficient) AND variation (e.g. standard deviation) or associated estimates of uncertainty (e.g. confidence intervals)
☐	☒	For null hypothesis testing, the test statistic (e.g. F, t, r) with confidence intervals, effect sizes, degrees of freedom and P value noted<br>*Give P values as exact values whenever suitable.*
☒	☐	For Bayesian analysis, information on the choice of priors and Markov chain Monte Carlo settings
☒	☐	For hierarchical and complex designs, identification of the appropriate level for tests and full reporting of outcomes
☐	☒	Estimates of effect sizes (e.g. Cohen's d, Pearson's r), indicating how they were calculated

Our web collection on statistics for biologists contains articles on many of the points above.

Software and code

Policy information about availability of computer code

Data collection	FACS: BD FACS Diva (BD Bioscience 6.4.1)<br>Microscopy: LAS X (Leica) and ZEN black (Carl Zeiss)
Data analysis	Image analysis: Fiji (v1.53c)<br>FACS: BD FACS Diva (BD Bioscience 6.4.1)<br>Statistics and plotting: python (3.8), scipy (1.5.3), pandas (1.3.5), numpy (1.23.5), matplotlib (3.7.1), and seaborn (0.12.2).<br>scRNAseq: scanpy (1.9.3), decoupler (PROGENY and DOROTHEA 1.4.0), scArches (0.5.9), scvelo (0.2.5), PANTHERdb (14.1)

For manuscripts utilizing custom algorithms or software that are central to the research but not yet described in published literature, software must be made available to editors and reviewers. We strongly encourage code deposition in a community repository (e.g. GitHub). See the Nature Portfolio guidelines for submitting code & software for further information.

Data

Policy information about availability of data

All manuscripts must include a data availability statement. This statement should provide the following information, where applicable:

- Accession codes, unique identifiers, or web links for publicly available datasets
- A description of any restrictions on data availability
- For clinical datasets or third party data, please ensure that the statement adheres to our policy

The generated scRNAseq data has been deposited in the Gene Expression Omnibus under the accession number (GEO ID to be confirmed). All other data that support the findings of this study are available from the corresponding author upon reasonable request.

Research involving human participants, their data, or biological material

Policy information about studies with human participants or human data. See also policy information about sex, gender (identity/presentation), and sexual orientation and race, ethnicity and racism.

Reporting on sex and gender	*Use the terms sex (biological attribute) and gender (shaped by social and cultural circumstances) carefully in order to avoid confusing both terms. Indicate if findings apply to only one sex or gender; describe whether sex and gender were considered in study design; whether sex and/or gender was determined based on self-reporting or assigned and methods used. Provide in the source data disaggregated sex and gender data, where this information has been collected, and if consent has been obtained for sharing of individual-level data; provide overall numbers in this Reporting Summary. Please state if this information has not been collected. Report sex- and gender-based analyses where performed, justify reasons for lack of sex- and gender-based analysis.*
Reporting on race, ethnicity, or other socially relevant groupings	*Please specify the socially constructed or socially relevant categorization variable(s) used in your manuscript and explain why they were used. Please note that such variables should not be used as proxies for other socially constructed/relevant variables (for example, race or ethnicity should not be used as a proxy for socioeconomic status). Provide clear definitions of the relevant terms used, how they were provided (by the participants/respondents, the researchers, or third parties), and the method(s) used to classify people into the different categories (e.g. self-report, census or administrative data, social media data, etc.) Please provide details about how you controlled for confounding variables in your analyses.*
Population characteristics	*Describe the covariate-relevant population characteristics of the human research participants (e.g. age, genotypic information, past and current diagnosis and treatment categories). If you filled out the behavioural & social sciences study design questions and have nothing to add here, write "See above."*
Recruitment	*Describe how participants were recruited. Outline any potential self-selection bias or other biases that may be present and how these are likely to impact results.*
Ethics oversight	*Identify the organization(s) that approved the study protocol.*

Note that full information on the approval of the study protocol must also be provided in the manuscript.

Field-specific reporting

Please select the one below that is the best fit for your research. If you are not sure, read the appropriate sections before making your selection.

☒ Life sciences ☐ Behavioural & social sciences ☐ Ecological, evolutionary & environmental sciences

For a reference copy of the document with all sections, see nature.com/documents/nr-reporting-summary-flat.pdf

Life sciences study design

All studies must disclose on these points even when the disclosure is negative.

Sample size	Required experimental sample sizes were estimated based on previous established protocols in the field. The sample sizes were adequate as the differences between experimental groups were reproducible. All n values are clearly indicated within the figure legends.
Data exclusions	No data was excluded from the analysis.
Replication	All animal experiments were performed by at least two independent researchers showing similar results. Ex vivo and in vitro experiments were replicated at least three times.
Randomization	Age- and weight-matched animals were randomly divided into treatment groups. Experiments that included both male and female mice were divided as to have same number of animals of the same sex in each group.
Blinding	No experiments presented in this study required blinding.

Reporting for specific materials, systems and methods

We require information from authors about some types of materials, experimental systems and methods used in many studies. Here, indicate whether each material, system or method listed is relevant to your study. If you are not sure if a list item applies to your research, read the appropriate section before selecting a response.

Materials & experimental systems

n/a	Involved in the study
☐	☒ Antibodies
☒	☐ Eukaryotic cell lines
☒	☐ Palaeontology and archaeology
☐	☒ Animals and other organisms
☒	☐ Clinical data
☒	☐ Dual use research of concern
☒	☐ Plants

Methods

n/a	Involved in the study
☒	☐ ChIP-seq
☐	☒ Flow cytometry
☒	☐ MRI-based neuroimaging

Antibodies

Antibodies used

Histology:
anti-PDPN (Abcam ab11936, 1:500 dilution)
anti-pSTAT3 (Cell Signaling Technology 9145S, 1:150)
anti-RUNX2 (Abcam ab92336, 1:150)
anti-GFP (Abcam ab13970, 1:500)
anti-PDGFRα (R&D systems AF1062, 1:100)
anti-KRT14 (Abcam ab181595, 1:100)
anti-PECAM1/CD31 (Abcam ab56299, 1:50)
anti-LYVE1 (Abcam ab14917, 1:100)
anti-αSMA (Abcam ab21027, 1:150)
anti-YAP1 (Abcam ab205270, 1:100)
anti-pSMAD2 (Cell Signalling 18338, 1:100)
anti-HIF1A (Novus NB100-479, 1:100)
anti-CCL2 (Abcam ab25124, 1:100)
anti-CXCL1 (R&D systems MAB453R, 1:100)
anti-ALDH1A3 (Novus NBP2-15339, 1:100)
anti-CYP26B1 (Elabscience E-AB-36196, 1:100)
anti-CD45/PTPRC (Abcam ab23910, 1:100)
anti-PI16 (R&D systems AF4929, 1:100)
FACS:
All antibodies were used in a 1:200 dilution except for the CD45 antibody, which was diluted 1:800.
anti-CD45(PTPRC)-APC and anti-CD45-PE/Cy7 (30-F11, Bio legend)
anti-CD31(PECAM1)-APC (390, e-Biosciences)
anti-TER119-APC (Ter119, Bio legend)
anti-CD326(EPCAM)-AF647 (G8.8, Bio legend)
anti-CD11b-AF488 (M1/70, Bio legend)
anti-LY6G-PacBlue (1A8, Bio legend)
anti-F4/80(ADGRE1)-APC (BM8, Bio legend)
anti-CD3-PE/Cy7 (500A2, Bio legend)
anti-CD19-BV510 (6D5, Bio legend)
anti-CD140a(PDGFRA)-PE-Cy7 (APA5, e-Biosciences).

Validation

See manufacturers' notes. Antibodies were additionally validated using respective isotype antibodies in immunofluorescence assays.

Animals and other research organisms

Policy information about studies involving animals; ARRIVE guidelines recommended for reporting animal research, and Sex and Gender in Research

Laboratory animals

Animal experiments were performed using 8- to 12-week-old adult mice.
C57BL/6J wildtype
En1tm2(cre)Wrst/J (En1Cre)
B6.Cg-Gt(ROSA)26Sortm14(CAG-tdTomato)Hze/J (R26Ai14)
B6.129(Cg)-Gt(ROSA)26Sortm4(ACTB-tdTomato.-EGFP)Luo/J (R26mTmG)
B6.129S6(Cg)-Gt(ROSA)26Sortm1(DTA)Jpmb/J (R26DTA)
PDPNCreER
CD201/ProcrCreER (Dr. Ariel Zeng, SIBCB)

B6.129-Hif1atm3Rsjo/J (Hif1aflox)

Wild animals	The study did not involve wild animals.
Reporting on sex	Male and female mice, equally distributed between time groups, were used for descriptive experiments. For functional studies, littermates or age-matched animals were randomly assigned into the different experimental groups. To decrease variability, only males were used for the RARγ agonist and Hif1α inhibition treatments (7 dpi), and for the Hif1α genetic deletion experiment. Females and males, equally distributed between treatment groups, were used for the PDPN+ cell ablation studies and for the 3 dpi Hif1α inhibition experiment.
Field-collected samples	The study did not involve samples collected from field.
Ethics oversight	Government of Upper Bavaria , Germany

Note that full information on the approval of the study protocol must also be provided in the manuscript.

Flow Cytometry

Plots

Confirm that:

☒ The axis labels state the marker and fluorochrome used (e.g. CD4-FITC).

☒ The axis scales are clearly visible. Include numbers along axes only for bottom left plot of group (a 'group' is an analysis of identical markers).

☒ All plots are contour plots with outliers or pseudocolor plots.

☒ A numerical value for number of cells or percentage (with statistics) is provided.

Methodology

Sample preparation	Skin and wounds were collected using 8 mm biopsy punch (Stiefel 10008) and fascia and dermis were separated as stated in the manuscript. Tissue was defatted, finely minced with scissors, and incubated in digestion solution (0.2 mg-1mg/ mL liberase, 0.5 mg/mL collagenase-A, 100 U/mL DNase in serum-free DMEM) for 60 minutes at 37C while rocking at 350 rpm. Digestion was stopped with 10 mL DMEM, samples were vortexed for few seconds, and then strained through 70 µm sieves. For the isolation of bone marrow cells, tibiae and femur of mice were dissected, flushed using a syringe with a G21 needle, and then filtered through 40µm filter to remove tissue debris. Single-cell suspensions were fixed with 2 % PFA to preserve surface markers stability for long periods. Single-cell suspensions were pelleted and suspended in PBS containing fluorophore-conjugated antibodies for 30 min before analysis.
Instrument	All samples were run on a BD LSRII cytometer (BD Biosciences) equipped with violet, blue, and red lasers and analysed in the BD FACS Diva analyser suite (BD Biosciences).
Software	BD FACS Diva (BD Bioscience 6.4.1)
Cell population abundance	The purity of sorted cells were determined by flow cytometric analysis of the sorted cells with the same gating strategy as during sorting
Gating strategy	For all cell types, initial gate from forward scatter (FSC-A) vs. side-scatter (SSC-A) plots was used. From this, single-cell gate from FSC-A vs FSC-H plots was used to exclude debris. Strict doublet exclusion was performed prior gating for immune cells (CD45+) and stromal cells (Lin-: CD45- CD31- Ter119- EpCAM-). Monocytes and macrophage cells were gated as ADGRE1+, Neutrophils as LY6G+, T-cells as CD3+, and B-cells as CD19+. Similarly, TdTomato+ cells from CD201CreERR26Ai14 mice were gated from Lin- cells then PDGFRA+, and from CD31+, CD45+, and EPCAM+ cells. Signal compensation was performed on FMO controls using compensation beads (Thermo Fisher 01-3333-42).

☒ Tick this box to confirm that a figure exemplifying the gating strategy is provided in the Supplementary Information.
